# A protocol to construct RNA-protein devices for photochemical translational regulation of synthetic mRNAs in mammalian cells

**DOI:** 10.1016/j.xpro.2022.101451

**Published:** 2022-06-10

**Authors:** Hideyuki Nakanishi, Tatsuyuki Yoshii, Shinya Tsukiji, Hirohide Saito

**Affiliations:** 1 Department of Life Science Frontiers, Center for iPS Cell Research and Application, Kyoto University, 53 Kawahara-cho, Shogoin, Sakyo-ku, Kyoto 606-8507, Japan; 2 Department of Biofunction Research, Institute of Biomaterials and Bioengineering, Tokyo Medical and Dental University (TMDU), 2-3-10 Kanda-Surugadai, Chiyoda-ku, Tokyo 101-0062, Japan; 3 Department of Life Science and Applied Chemistry, Nagoya Institute of Technology, Gokiso-cho, Showa-ku, Nagoya 466-8555, Japan; 4 PRESTO, Japan Science and Technology Agency (JST), 4-1-8 Honcho, Kawaguchi, Saitama 332-0012, Japan; 5 Department of Nanopharmaceutical Sciences, Nagoya Institute of Technology, Gokiso-cho, Showa-ku, Nagoya 466-8555, Japan

**Keywords:** Flow Cytometry/Mass Cytometry, Gene Expression, Biotechnology and bioengineering, Chemistry

## Abstract

Here, we describe a protocol for the translational regulation of transfected messenger RNAs (mRNAs) using light in mammalian cells. We detail the steps for photocaged ligand synthesis, template DNA preparation, and mRNA synthesis. We describe steps for mRNA transfection, treatment of cells with a photocaged ligand followed by light irradiation, and analysis of the transgene expression. The protocol enables spatiotemporally regulated transgene expression without the risk of insertional mutagenesis.

For complete details on the use and execution of this protocol, please refer to Nakanishi et al. (2021).

## Highlights

• Synthesis of photocaged ligands necessary for photo-regulation of mRNA translation
• Design and construction of template DNAs for *in vitro* transcription (IVT) of mRNAs
• Preparation of mRNAs by IVT for transgene expression in mammalian cells
• Photochemical translational regulation after mRNA transfection into mammalian cells

## Abstract (editor highlights)

Publisher’s note: Undertaking any experimental protocol requires adherence to local institutional guidelines for laboratory safety and ethics.

## Graphical abstract

**Figure:**
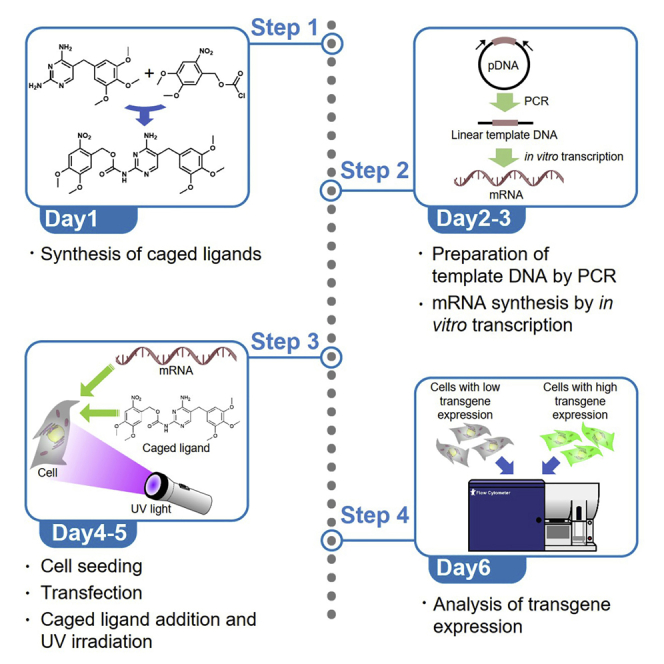

## Before you begin

### Selection of reporter genes to be regulated

**Timing: 1 h**

1. If you will use a flow cytometer to analyze the translational activation or repression of the target mRNAs, check the laser-filter sets of the flow cytometer and select fluorescent proteins with excitation and emission wavelength peaks that are close to those available on the flow cytometer. Not only excitation and emission wavelength but also other properties such as brightness and cytotoxicity should also be considered. This may be eased by using a fluorescent protein database, such as FPbase (Lambert, 2019).
2. Alternatively, if you will use a luminometer, you can use luciferase genes as reporters.

### Design of primers

**Timing: 1 h**

**Figure 1:**
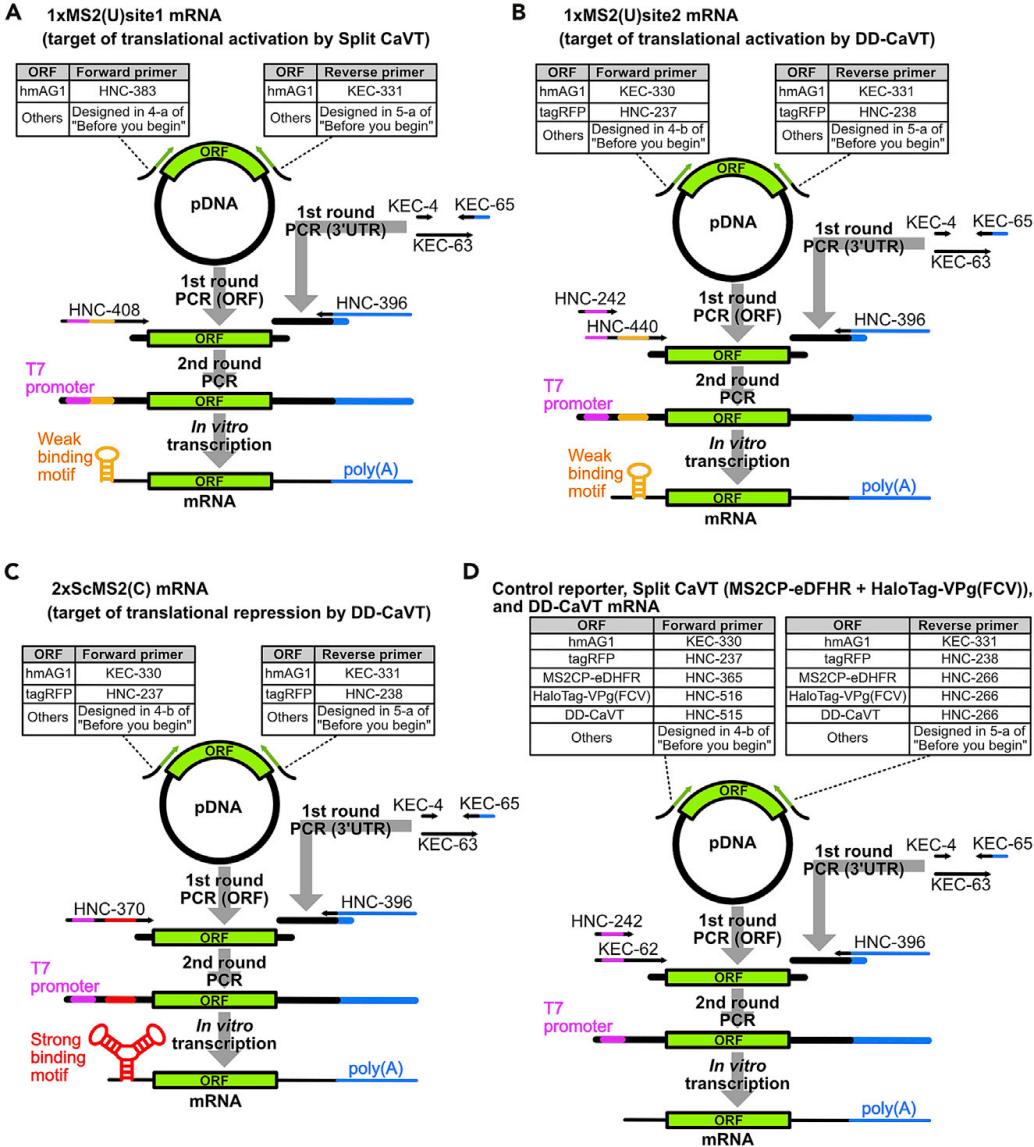
Scheme to prepare mRNAs by in vitro transcription (IVT) First, template DNAs for IVT which contain T7 promoter are prepared by two rounds of PCR using the indicated primers. Then, mRNAs are transcribed from the template DNAs by T7 RNA polymerase. (A) 1x MS2(U)site1 mRNA, a target of translational activation by Split CaVT. (B) 1xMS2(U)site2 mRNA, a target of translational activation by DD-CaVT. (C) 2xScMS2(C) mRNA, a target of translational repression by DD-CaVT. (D) Other mRNAs.

If using the fluorescent proteins hmAG1 and tagRFP as the target of translational regulation and control reporter, respectively, all the necessary primers to prepare the in vitro transcription (IVT) template DNAs are listed in the key resources table. Otherwise, some primers need to be designed as follows (Figure 1).

3. Design forward and reverse primers to amplify the translational regulation-target gene (or control reporter gene) by PCR from a source template (e.g., plasmid DNA (pDNA)). Primer design tools, such as Primer3Plus (Untergasser et al., 2007), may be helpful.

***Note:*** The amplified sequence should begin with a Kozak sequence including the start codon and end with a stop codon. For clarity, these features are included directly in the steps below, but alternatively, if they are present in the template DNA they can be amplified directly instead.

4. Add the appropriate partial 5′ untranslated region (UTR) sequence (underlined) to the 5′ end of the forward primer designed in step 3 (represented by NNN…) along with a Kozak sequence including the start codon (**bold**).
  a. For 1xMS2(U)site1 mRNA.
    AGAAAAGAAGAGTAAGAAGAAATATAAGACACCGGTC**GCCACCATG**NNN…
  b. For other mRNAs (1xMS2(U)site2, 2xScMS2(C), and a control reporter).
    CACCGGTC**GCCACCATG**NNN…
5. Add the appropriate partial 3′ UTR (underlined) to the reverse primer designed in step 3 (represented by NNN…) and a reverse-complement stop codon (**bold**).
  a. GCCCCGCAGAAGGTCTAGAT**TCA**NNN…

***Note:*** It depends on the type of Caliciviral VPg-based Translational activator (CaVT) whether 1xMS2(U)site1 or site2 mRNA is preferable for the translational activation. We previously showed that 1xMS2(U)site2 mRNA is preferable for non-split type CaVT-mediated translational activation. On the other hand, when split CaVT is used, 1xMS2(U)site1 mRNA is preferable (Nakanishi and Saito, 2020). However, both types of mRNAs can be translationally activated by both types of CaVT.

***Note:*** We adopt the procedure of adding part of the UTRs in the 1^st^ round PCR and the rest of the UTRs and T7 promoter in the 2^nd^ round PCR because long primers are expensive or cannot be ordered. However, if ordering long primers is not a problem, it is also possible to prepare template DNAs directly from pDNAs by a single PCR.

## Key resources table

**Table:** 

REAGENT or RESOURCE	SOURCE	IDENTIFIER
**Bacterial and virus strains**		

*E. coli*: HST08 strain	Takara Bio	9128

**Chemicals, peptides, and recombinant proteins**		

Acetonitrile (MeCN, HPLC grade)	Kanto Chemical	01033-5B
Boc-8-amino-3,6-dioxaoctanoic acid (dicyclohexylamine salt)	Watanabe Chemical	M01867
*O*-(Benzotriazole-1-yl)-*N*,*N*,*N*,*N′*-tetramethyluronium hexafluorophosphate (HBTU)	Watanabe Chemical	A00149
Chloroform (CHCl_3_)	Kanto Chemical	07278-80
2-(2-((6-Chlorohexyl)oxy)ethoxy)ethan-1-amine	(Zhang et al., 2006)	N/A
Citric acid	FUJIFILM Wako Pure Chemicals	030-05525
5-(4-((2,4-Diaminopyrimidine-5-yl)methyl)-2,6-dimethoxyphenoxy)pentanoic acid	(Ando et al., 2007)	N/A
Dichloromethane (CH_2_Cl_2_)	Kanto Chemical	10158-70
Dichloromethane (CH_2_Cl_2_, dehydrated)	Kanto Chemical	11338-05
4,5-Dimethoxy-2-nitrobenzyl chloroformate	Merck	420069-1G
*N*,*N*-Dimethylformamide (DMF, dehydrated)	Kanto Chemical	11339-05
*N*,*N*-Diisopropylethylamine (DIPEA)	Watanabe Chemical	A00030
Ethyl acetate (EtOAc)	Kanto Chemical	14029-80
Ethyl 5-(4-((2,4-diaminopyrimidine-5-yl)methyl)-2,6-dimethoxyphenoxy)pentanoate	(Ando et al., 2007)	N/A
1-hydroxybenzotriazole (monohydrate) (HOBt·H_2_O)	Watanabe Chemical	A00014
Hydrochloric acid (HCl, 6 N)	Kanto Chemical	18588-08
LiOH	TCI	L0225
Methanol (MeOH)	Kanto Chemical	25183-80
MgSO_4_ (anhydrous)	Kanto Chemical	25035-00
NaCl	FUJIFILM Wako Pure Chemicals	195-01663
NaHCO_3_	FUJIFILM Wako Pure Chemicals	191-01305
Na_2_SO_4_ (anhydrous)	FUJIFILM Wako Pure Chemicals	199-03344
Silica gel	Kanto Chemical	37563-84
Toluene	Kanto Chemical	40180-70
Trifluoroacetic acid (TFA)	Watanabe Chemical	A00026
Trimethoprim (TMP)	TCI	T2286
PrimeSTAR Max DNA Polymerase	Takara Bio	R045A
Tris-Acetate-EDTA buffer (50×)	Nacalai Tesque	32666-81
Agarose S	Nippon Gene	312-01193
Quick-Load Purple 1 kb Plus DNA Ladder	New England Biolabs	N0550
Midori Green Advance	Nippon Genetics	MG04
DpnI	Toyobo	DPN-101
ARCA (Anti Reverse Cap Analog)	TriLink	N-7003-10
G(5′)ppp(5′)A RNA Cap Structure Analog	New England Biolabs	S1406L
N^1^-Methylpseudo-UTP	TriLink	N-1081-10
MEGAscript T7 transcription kit	Thermo Fisher Scientific	AMB13345
rAPid Alkaline Phosphatase	Roche	4898133001
DMEM(4.5 g/L Glucose) with L-Gln, without Sodium Pyruvate, liquid ∗If using cells other than HeLa, use an appropriate medium.	Nacalai Tesque	08459-64
Antibiotic Antimycotic Solution ∗If using cells other than HeLa, use an appropriate antibiotic.	Sigma-Aldrich	A5955
MEM Non-Essential Amino Acids Solution, 100× ∗If using cells other than HeLa, use an appropriate medium supplement.	Thermo Fisher Scientific	11140-050
Sodium pyruvate solution, 100 mM ∗If using cells other than HeLa, use an appropriate medium supplement.	Sigma-Aldrich	S8636
Trypsin-EDTA (0.25%), phenol red	Thermo Fisher Scientific	25200072
Opti-MEM Reduced Serum Medium	Thermo Fisher Scientific	31985-070
Lipofectamine MessengerMAX Transfection Reagent	Thermo Fisher Scientific	LMRNA008

**Experimental models: Cell lines**		

HeLa (human cervical carcinoma cell)	ATCC	ATCC Cat# CCL-2, RRID:CVCL_0030

**Oligonucleotides**		

HNC-237 (CACCGGTCGCCACCATGGTGTCTAAGG GCGAAGAGCTGA) ∗Only when using tagRFP as a target or control reporter gene	Eurofins Genomics	N/A
HNC-238 (GCCCCGCAGAAGGTCTAGATTCAATTA AGTTTGTGCCCCAGTTTG) ∗Only when using tagRFP as a target or control reporter gene	Eurofins Genomics	N/A
HNC-242 (CAGTGAATTGTAATACGACTCACTATAGGGCGA)	Eurofins Genomics	N/A
HNC-266 (GCCCCGCAGAAGGTCTAGATTCACTTA TCGTCGTCATCCTTG)	Eurofins Genomics	N/A
HNC-365 (CACCGGTCGCCACCATGGCTTCTAACTTTAC)	Eurofins Genomics	N/A
HNC-370 (CAGTGAATTGTAATACGACTCACTATA GGGTCAGATCCGCTAGCGGATCCGGGAGCAGG TGAGGATCACCCATCTGCCACGAGCGAGGTGA GGATCACCCATCTCGCTCGTGTTCCCACCGGTC GCCACCATG)	Eurofins Genomics	N/A
HNC-383 (AGAAAAGAAGAGTAAGAAGAAATATA AGACACCGGTCGCCACCATGGTGAGCGTGATC AAGCCCGAGA) ∗Only when using hmAG1 as a target reporter gene	Eurofins Genomics	N/A
HNC-396 (TTTTTTTTTTTTTTTTTTTTTTTTTTTTTTT TTTTTTTTTTTTTTTTTTTTTTTTTTTTTTTTTTTTTTT TTTTTTTTTTTTTTTTTTTTTTTTTTTTTTTTTTTTTTT TTTTTTTTTTTCCTACTCAGGCTTTATTCA)	Eurofins Genomics	N/A
HNC-408 (CAGTGAATTGTAATACGACTCACTATA GGGACATGAGGATTACCCATGTCGAATTAAGAG AGAAAAGAAGAGTAAGAAGAAATATAAGACACC)	Eurofins Genomics	N/A
HNC-440 (TGTAATACGACTCACTATAGGGCGAATTA AGAGAGAAAAGAAGAGTACATGAGGATTACCCATG TAAGAAGAAATATAAGACACCGGTCGCCACCATG)	Eurofins Genomics	N/A
HNC-515 (CACCGGTCGCCACCATGATCAGTCTGATTGC)	Eurofins Genomics	N/A
HNC-516 (CACCGGTCGCCACCATGGCAGAAATCGGTA)	Eurofins Genomics	N/A
KEC-4 (TCTAGACCTTCTGCGGGGC)	Eurofins Genomics	N/A
KEC-62 (CAGTGAATTGTAATACGACTCACTAT AGGGCGAATTAAGAGAGAAAAGAAGAGTAA GAAGAAATATAAGACACCGGTCGCCACCATG)		
KEC-63 (TCTAGACCTTCTGCGGGGCTTGCCTTC TGGCCATGCCCTTCTTCTCTCCCTTGCACCTGT ACCTCTTGGTCTTTGAATAAAGCCTGAGTAGG)	Eurofins Genomics	N/A
KEC-65 (TTTTTTTTTTTTTTTTTTTTCCTACTCAGG CTTTATTCAAAGACCAAG)	Eurofins Genomics	N/A
KEC-330 (CACCGGTCGCCACCATGGTGAGCGT GATCAAGCCCG) ∗Only when using hmAG1 as a target or control reporter gene	Eurofins Genomics	N/A
KEC-331 (GCCCCGCAGAAGGTCTAGATTCAC TTGGCCTGGCTGGGC) ∗Only when using hmAG1 as a target or control reporter gene	Eurofins Genomics	N/A

**Recombinant DNA**		

pcDNA3.1-MS2CP-VPg(FCV)	Addgene	#167314
pBCMV-MS2CP-eDHFR	Addgene	#167309
pBCMV-HaloTag-VPg(FCV)	Addgene	#167311
pcDNA3.1-ecDHFR(DD)-MS2CP-VPg(FCV)	Addgene	#167313
pFucci-S/G2/M Green ∗Only when using hmAG1 as a target or control reporter gene	MBL	AM-V9014M
pTagRFP-actin ∗Only when using tagRFP as a target or control reporter gene	Evrogen	FP144

**Software and algorithms**		

FlowJo (optional)	Becton, Dickinson and Company	https://www.flowjo.com/solutions/flowjo

**Other**		

ProFlex PCR System (or a comparable thermal cycler)	Thermo Fisher Scientific	4484073
Mupid-2plus (or a comparable electrophoresis apparatus)	Mupid	M-2P
Gel Doc EZ (or a comparable gel imager)	Bio-Rad	1708270
MX-305 (or a comparable centrifuge)	Tomy	MX-305
NanoDrop 2000 (or a comparable spectrophotometer)	Thermo Fisher Scientific	ND-2000
Agilent 2100 Bioanalyzer	Agilent Technologies	G2939BA
HP-30LM UV lamp	Atto	HP-30LM
BD Accuri C6 Plus (or a comparable flow cytometer)	BD Biosciences	660517

PureYield Plasmid Miniprep System	Promega	A1222
Monarch PCR & DNA Cleanup Kit	New England Biolabs	T1030L
Monarch RNA Cleanup Kit	New England Biolabs	T2050L
Agilent RNA 6000 pico kit	Agilent technologies	5067-1513
Falcon cell strainer 35 μm	Corning	352235
DNA LoBind 1.5 mL Tube	Eppendorf	0030108051

## Step-by-step method details

A photocaged ligand (photocaged Trimethoprim-HaloTag ligand (TMP-HL) for split CaVT and photocaged trimethoprim (TMP) for destabilizing domain-fused CaVT (DD-CaVT), respectively) is necessary for photochemical translational regulation of synthetic mRNAs by split CaVT or DD-CaVT.

### Synthesis of TMP-HL (**1**)

**Timing: 16 h for synthesis of compound 4**

**Timing: 10 h synthesis of TMP-HL (1)**

**CRITICAL:** The synthesis scales do not always match between steps, but these scales have been optimized and changing them may result in reduced yields.

**Figure 2:**
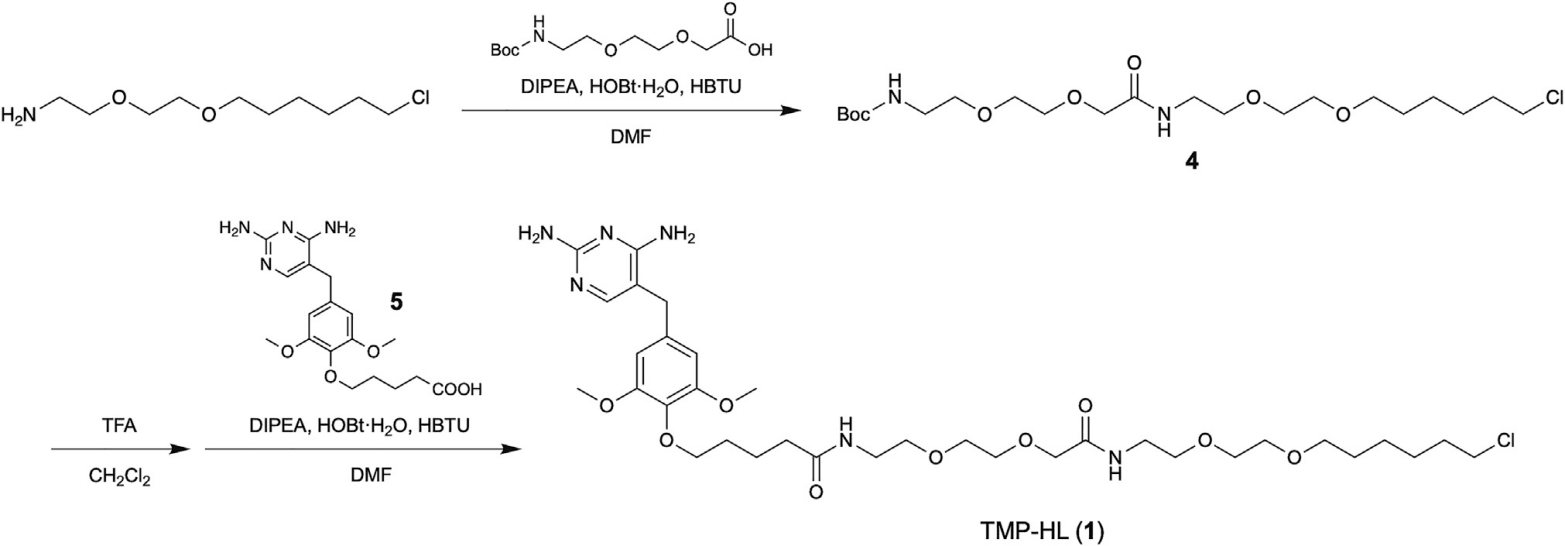
Synthetic scheme of TMP-HL (**1**)

The following steps describe the synthesis and characterization of TMP-HL (**1**), see Figure 2.

***Note:*** All the procedures should be conducted in a fume food. Unless noted all rotary evaporation steps are carried out at 25°C.

1. Synthesis of compound **4**.
  a. Weigh 227 mg (1.24 mmol) of 2-(2-((6-chlorohexyl)oxy)ethoxy)ethan-1-amine (Zhang et al., 2006; Singh et al., 2013) in a 50 mL two-neck round-bottom flask containing a magnetic stirring bar. Equip the flask with an argon-filled balloon.
  b. Add 10 mL of dry dimethylformamide (DMF), 400 mg (0.90 mmol) of Boc-8-amino-3,6-dioxaoctanoic acid (dicyclohexylamine salt), 783 μL (4.60 mmol) of N,N-Diisopropylethylamine (DIPEA), 165 mg (1.08 mmol) of HOBt·H_2_O, and 409 mg (1.08 mmol) of 1-[Bis(dimethylamino)methylene]-1H-benzotriazolium 3-oxide hexafluorophosphate (HBTU).
  c. Stir the reaction mixture at room temperature (20°C–25°C) for 8 h under argon.
  d. Remove the solvent using a rotary evaporator under reduced pressure at 40°C.
  e. Dissolve the crude mixture in 50 mL of EtOAc. Wash the organic layer with 5% aqueous citric acid solution (30 mL × 3), saturated aqueous NaHCO_3_ solution (30 mL × 3), and brine (30 mL × 1) in a separatory funnel. Collect the organic layer and dry it with anhydrous Na_2_SO_4_ (10 g). After filtration to remove Na_2_SO_4_, condense the organic layer using a rotary evaporator under reduced pressure at 35°C until the solvent is no longer detectable.
  f. Purify the crude product using silica gel column chromatography (100 mL bed volume, washed with 150 mL CHCl_3_, and 100 mL 30:1 CHCl_3_/MeOH, then eluted with 300 mL 30:1 CHCl_3_/MeOH).
  g. Collect the fractions and remove solvent using a rotary evaporator at 30°C.
  h. Dry the sample under reduced pressure at RT for at least 1 h to afford compound **4** (413 mg, yield 98%) as a colorless oil.
  i. Characterize the product by ^1^H NMR spectroscopy. ^1^H-NMR (400 MHz, CDCl_3_):δ [ppm] 5.25 (1H, brs), 4.01 (2H, s), 3.67 (2H, m), 3.64–3.61 (4H, m), 3.59–3.50 (10H, m), 3.45 (2H, t, *J* = 6.6 Hz), 3.33 (2H, m), 1.77 (2H, m), 1.59 (2H, m), 1.45 (9H, s), 1.41–1.35 (4H, m).

**Pause point:** At this point, the product can be stored at –20°C for at least 2 years.

2. Synthesis of TMP-HL (**1**).
  a. Weigh 49 mg (0.104 mmol) of compound **4** in a 50 mL two-neck round-bottom flask containing a magnetic stirring bar.
  b. Add 1 mL of CH_2_Cl_2_ and 0.5 mL of trifluoroacetic acid (TFA).
  c. Stir the reaction mixture at room temperature for 1 h.
  d. Add 1 mL of toluene to the mixture and remove the solvent using a rotary evaporator under reduced pressure at 40°C. Repeat this process two more times to afford a deprotected form of compound **4** as a colorless oil.
  e. Equip the flask with an argon-filled balloon.
  f. Dissolve the crude product with 2 mL of anhydrous DMF.
  g. Add 31.2 mg (0.083 mmol) of 5-(4-((2,4-diaminopyrimidin-5-yl)methyl)-2,6-dimethoxyphenoxy)pentanoic acid (compound **5**) (Ando et al., 2007; Cai et al., 2019), 54.3 μL (0.319 mmol) of DIPEA, 15.9 mg (0.104 mmol) of HOBt·H_2_O, and 39.4 mg (0.104 mmol) of HBTU.
  h. Stir the reaction mixture at room temperature for 2 h under argon.
  i. Remove the solvent using a rotary evaporator under reduced pressure at 35°C.
  j. Purify the crude product using silica gel column chromatography (100 mL bed volume, washed with 50 mL CHCl_3_, 100 mL 50:1 CHCl_3_/MeOH, and 100 mL 20:1 CHCl_3_/MeOH, then eluted with 800 mL 10:1 CHCl_3_/MeOH).
  k. Collect the fractions and remove solvent using a rotary evaporator at 30°C.
  l. Dry the sample under reduced pressure at RT for at least 1 h to afford TMP-HL (**1**) (14.2 mg, yield 24%) as a colorless wax.
  m. Characterize the product by ^1^H NMR spectroscopy and ESI-MS. ^1^H-NMR (400 MHz, CD_3_OD): δ [ppm] 7.36 (1H, s), 6.54 (2H, s), 3.98 (2H, s), 3.91 (2H, m), 3.79 (6H, s), 3.68–3.62 (6H, m), 3.60–3.53 (10H, m), 3.50–3.36 (6H, m), 2.29 (2H, t, *J* = 7.2 Hz), 1.88–1.65 (6H, m), 1.65–1.53 (2H, m), 1.49–1.34 (4H, m). HRMS (ESI): calculated for [M+H]+, 727.3792; found, 727.3771.

**Pause point:** The product can be stored at –20°C for at least 2 years.

### Synthesis of photocaged TMP-HL (**2**)

**Timing: 19 h for synthesis of compound 7**

**Timing: 10 h for synthesis of compound 8**

**Timing: 18 h for synthesis of photocaged TMP-HL (2)**

**Figure 3:**
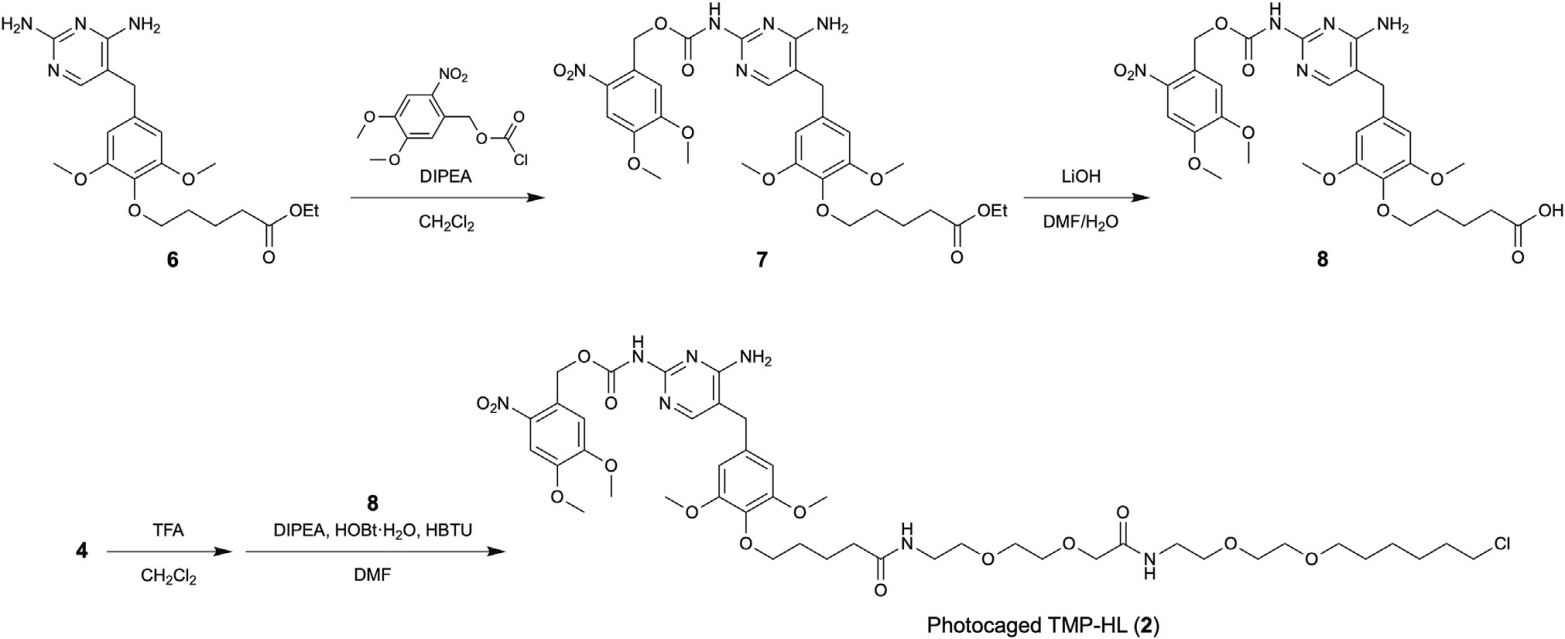
Synthetic scheme of photocaged TMP-HL (**2**)

The following steps describe the synthesis and characterization of photocaged TMP-HL (**2**), see Figure 3.

***Note:*** All the procedures should be operated in a fume food.

3. Synthesis of compound **7**.
  a. Weigh 100 mg (0.25 mmol) of ethyl 5-(4-((2,4-diaminopyrimidin-5-yl)methyl)-2,6-dimethoxyphenoxy)pentanoate (compound **6**) (Ando et al., 2007; Cai et al., 2019) in a 50 mL two-neck round-bottom flask containing a magnetic stirring bar. Equip the flask with an argon-filled balloon.
  b. Add 2.5 mL of dry CH_2_Cl_2_ and 43 μL (0.25 mmol) of DIPEA.
  c. Stir the solution and cool the flask to 0°C in an ice-water bath.
  d. Slowly add 68 mg (0.25 mmol) of 4,5-dimethoxy-2-nitrobenzyl chloroformate at 0°C to the flask.
  e. Stir the reaction mixture at room temperature for 9 h under argon.
  f. Dilute the reaction mixture with 20 mL of CH_2_Cl_2_ and wash the organic layer with water (20 mL × 2) and brine (20 mL × 1) in a separatory funnel. Collect the organic layer and dry it with anhydrous Na_2_SO_4_. After filtration to remove Na_2_SO_4_, condense the organic layer until the solvent is no longer detectable using a rotary evaporator under reduced pressure.
  g. Purify the crude product using silica gel column chromatography (100 mL bed volume, washed with 200 mL CHCl_3_, 600 mL 20:1 CHCl_3_/MeOH, then eluted with 800 mL 50:1 CHCl_3_/MeOH)).
  h. Collect the fractions and remove solvent using rotary evaporator at 30°C.
  i. Dry the sample under reduced pressure at RT for at least 1 h to afford compound **7** (18 mg, yield 11%) as a pale yellow solid.
  j. Characterize the product by ^1^H NMR spectroscopy. ^1^H-NMR (400 MHz, DMSO-*d*_*6*_): δ [ppm] 10.01 (1H, brs), 7.78 (1H, s), 7.73 (1H, s), 7.46 (1H, s), 6.67 (2H, brs), 6.59 (2H, s), 5.43(2H, s), 4.05 (2H, q, *J* = 7.2 Hz), 3.92 (3H, s), 3.87 (3H, s), 3.78 (2H, t, *J* = 6.0 Hz), 3.71 (6H, s), 3.63 (2H, s), 2.34 (2H, t, *J* = 7.4 Hz), 1.71–1.58 (4H, m), 1.17 (3H, t, *J* = 7.2 Hz). The spectral data should be in agreement with the 2′-amino-caged TMP structure previously reported (Ballister et al., 2014).

**Pause point:** At this point, the product can be stored at –20°C for at least 2 years.

4. Synthesis of compound **8**.
  a. Weigh 100 mg (0.16 mmol) of compound **7** in a 50 mL two-neck round-bottom flask containing a magnetic stirring bar.
  b. Add 10 mL of DMF to the reaction flask and stir the solution at room temperature.
  c. Add 3 mL (3.0 mmol) of 1 M aqueous LiOH solution and stir the reaction mixture at room temperature for 2 h.
  d. Neutralize the reaction mixture with 0.5 mL of 6 M aqueous HCl solution.
  e. Remove the solvent using a rotary evaporator under reduced pressure.
  f. Dissolve the crude mixture with 30 mL of 0.1 M aqueous NaOH solution and wash the aqueous layer with 30 mL CH_2_Cl_2_ three times. Collect the aqueous layer.
  g. Acidify the aqueous layer to ca. pH 4 with 6 M aqueous HCl solution to form a precipitate.
  h. Collect the precipitate by filtration and dry it under reduced pressure to afford compound **8** (75 mg, yield 71%) as a pale yellow solid.
  i. Characterize the product by ^1^H NMR spectroscopy. ^1^H-NMR (400 MHz, DMSO-*d*_*6*_): δ [ppm] 12.0 (1H, brs), 9.99 (1H, s), 7.78 (1H, s), 7.73 (1H, s), 7.46 (1H, s), 6.65 (2H, brs), 6.59 (2H, s), 5.43 (2H, s), 3.92 (3H, s), 3.87 (3H, s), 3.78 (2H, t, *J* = 5.8 Hz), 3.71 (6H, s), 3.63 (2H, s), 2.26 (2H, t, *J* = 6.8 Hz), 1.71–1.58 (4H, m).

**Pause point:** At this point, the product can be stored at –20°C for at least 2 years.

5. Synthesis of photocaged TMP-HL (**2**).
  a. Weigh 12.6 mg (27 μmol) of compound **4** in a 50 mL two-neck round-bottom flask containing a magnetic stirring bar.
  b. Add 2 mL of CH_2_Cl_2_ and 2 mL of TFA.
  c. Stir the reaction mixture at room temperature for 1 h.
  d. Add 1 mL of toluene to the mixture and remove the solvent using a rotary evaporator under reduced pressure. Repeat this process two more times to afford a deprotected form of compound **4** as a colorless oil.
  e. Equip the flask with an argon-filled balloon.
  f. Dissolve the crude product with 1 mL of dry DMF.
  g. Add 11 mg (18 μmol) of compound **8**, 31 μL (182 μmol) of DIPEA, 4.1 mg (27 μmol) of HOBt·H_2_O, and 10.2 mg (27 μmol) of HBTU.
  h. Stir the reaction mixture at room temperature for 6 h under argon.
  i. Remove the solvent using a rotary evaporator under reduced pressure.
  j. Dissolve the crude product with 20 mL of EtOAc and wash the organic layer with 0.1 M aqueous HCl solution (20 mL × 1) and saturated NaHCO_3_ solution (20 mL × 1) in a separatory funnel. Collect the organic layer and dry it with anhydrous Na_2_SO_4_. After filtration to remove Na_2_SO_4_, condense the organic layer using a rotary evaporator under reduced pressure at 35°C.
  k. Purify the crude product by reversed-phase HPLC using a semi-preparative C18 column (a linear gradient of MeCN containing 0.1% TFA and 0.1% aqueous TFA solution) to afford photocaged TMP-HL (**2**) (9.5 mg, yield 55%) as a white solid after lyophilization.
  l. Characterize the product by ^1^H NMR spectroscopy and ESI-MS. ^1^H-NMR (400 MHz, CD_3_OD): δ [ppm] 7.78 (1H, s), 7.41 (1H, s), 7.27 (1H, s), 6.60 (2H, s), 5.66 (2H, s), 3.98 (5H, m), 3.95–3.91 (5H, m), 3.81 (6H, s), 3.74 (2H, s), 3.68–3.63 (4H, m), 3.61–3.52 (10H, m), 3.48–3.39 (6H, m), 2.29 (2H, t, *J* = 7.2 Hz), 1.82–1.70 (6H, m), 1.61–1.54 (2H, m), 1.48–1.34 (4H, m). HRMS (ESI): calculated for [M+H]+, 966.4222; found, 966.4179.

**Pause point:** The product can be stored at –20°C for at least 2 years.

### Synthesis of photocaged TMP (3)

**Timing: 14 h**

**Figure 4:**
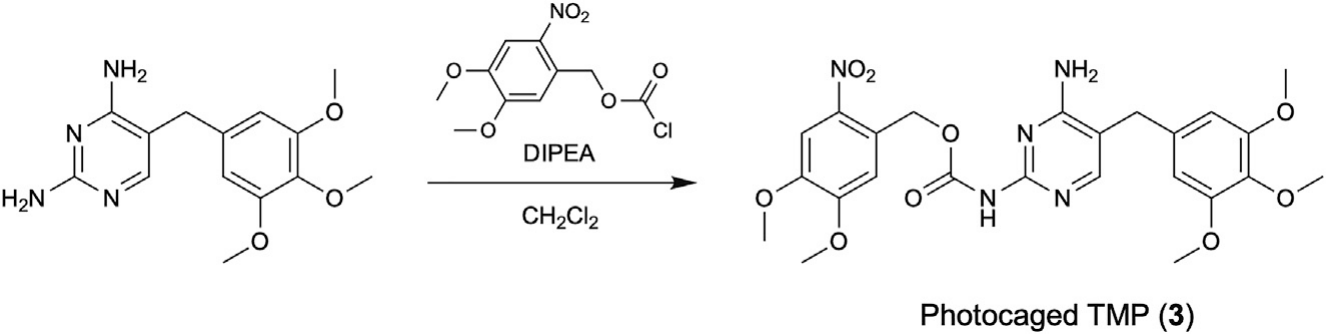
Synthetic scheme of photocaged TMP (**3**)

The following steps describe the synthesis and characterization of photocaged TMP (**3**), see Figure 4.

***Note:*** All the procedures should be operated in a fume food.

6. Synthesis of photocaged TMP (**3**).
  a. Weigh 157 mg (0.54 mmol) of trimethoprim in a 50 mL two-neck round-bottom flask containing a magnetic stirring bar. Equip the flask with an argon-filled balloon.
  b. Add 3 mL of dry CH_2_Cl_2_ and 64.8 μL (0.38 mmol) of DIPEA.
  c. Stir the solution and cool the flask to 0°C on an ice-water bath.
  d. Slowly add 100 mg (0.36 mmol) of 4,5-dimethoxy-2-nitrobenzyl chloroformate at 0°C to the flask.
  e. Stir the reaction mixture at room temperature for 5 h under argon.
  f. Dilute the reaction mixture with 40 mL of CH_2_Cl_2_ and wash the organic layer with 40 mL water once and then 40 mL brine once in a separatory funnel. Collect the organic layer and dry it with anhydrous MgSO_4_. After filtration to remove MgSO_4_, condense the organic layer using a rotary evaporator under reduced pressure until the solvent is no longer detectable.
  g. Purify the crude product by silica gel column chromatography 100 mL bed volume, washed with 900 mL CHCl_3_, and eluted with 900 mL 100:1 CHCl_3_/MeOH).
  h. Collect the fractions and remove solvent using rotary evaporator at 30°C.
  i. Dry the sample under reduced pressure at RT for at least 1 h to afford photocaged TMP (**3**) (14.3 mg, yield 7%) as a pale yellow solid.
  j. Characterize the product by ^1^H NMR spectroscopy and ESI-MS. ^1^H-NMR (400 MHz, DMSO-*d*_*6*_): δ [ppm] 10.02 (1H, s), 7.79 (1H, s), 7.73 (1H, s), 7.46 (1H, s), 6.67 (2H, brs), 6.60 (2H, s), 5.43 (2H, s), 3.92 (3H, s), 3.88 (3H, s), 3.73 (6H, s), 3.63 (2H, s), 3.62 (3H, s). The spectral data should be in agreement with the 2′-amino-caged TMP structure previously reported (Ballister et al., 2014). HRMS (ESI): calculated for [M+H]^+^, 530.1882; found, 530.1886.

**Pause point:** The product can be stored at –20°C for at least 2 years.

### Preparation of template DNAs for IVT

**Timing: 3–4 h**

Template DNAs for IVT are prepared by two rounds of PCR (Figure 1). Two types of DNA fragments are obtained in the 1^st^ round. One is the DNA containing the translational regulation-target or CaVT gene (hereafter called open reading frames (ORF)) flanked with the partial 5′ and 3′ UTR sequences. The other is the DNA containing the 3′ UTR sequence. In the 2^nd^ round of PCR, these two DNA fragments are fused and the sequences of T7 promoter, 5′ UTR, and poly(A) tail are added. Although the protocol uses PrimeSTAR Max DNA polymerase, this can be substituted with another high-fidelity PCR enzyme following the manufacturer’s recommended protocol.

7. 1^st^ round PCR.
  a. To amplify the ORF flanked with the partial 5′ and 3′ UTR sequences, prepare the PCR mixtures shown below.

**Table:** 

Reagent	Final concentration	Amount
PrimeSTAR Max Premix (2×)	1×	12.5 μL
10 μM Forward primer	0.3 μM	0.75 μL
10 μM Reverse primer	0.3 μM	0.75 μL
Template pDNA	0.02 ng/μL	0.5 ng (variable volume)
dH_2_O	n/a	Dilute to 25 μL
**Total**	n/a	25 μL
    To find appropriate primers and template DNAs, refer to the following list and the key resources table.

**Table:** 

ORF name	Forward primer	Reverse primer	Template pDNA
ORF for 1xMS2(U)site1	Designed in 4-a of “before you begin”. ∗In the case of hmAG1, you can use HNC-383.	Designed in 5-a of “before you begin”. ∗In the case of hmAG1, you can use KEC-331.	pDNA containing the gene to be regulated ∗In the case of hmAG1, you can use pFucci-S/G2/M Green.
ORF for 1xMS2(U)site2 and 2xScMS2(C), and control reporter	Designed in 4-b of “before you begin”. ∗In the case of hmAG1 and tagRFP, you can use KEC-330 and HNC-237, respectively.	Designed in 5-a of “before you begin”. ∗In the case of hmAG1 and tagRFP, you can use KEC-331 and HNC-238, respectively.	pDNA containing the gene to be regulated or control reporter gene. ∗In the case of hmAG1 and tagRFP, you can use pFucci-S/G2/M Green and pTagRFP-actin, respectively.
MS2CP-eDHFR (N-terminal fragment of split CaVT)	HNC-365	HNC-266	pBCMV-MS2CP-eDHFR
HaloTag-VPg(FCV) (C-terminal fragment of split CaVT)	HNC-516	HNC-266	pBCMV-HaloTag-VPg(FCV)
DD-CaVT	HNC-515	HNC-266	pcDNA3.1-ecDHFR(DD)-MS2CP-VPg(FCV)
CaVT	HNC-365	HNC-266	pcDNA3.1-MS2CP-VPg(FCV)
  b. To amplify DNA containing the 3′ UTR sequence, prepare the PCR mixture shown below. The primer sequences are shown in the key resources table.

**Table:** 

Reagent	Final concentration	Amount
PrimeSTAR Max Premix (2×)	1×	25 μL
10 μM KEC-4	0.3 μM	1.5 μL
10 μM KEC-65	0.3 μM	1.5 μL
10 nM KEC-63 (template DNA)	0.3 nM	1.5 μL
dH_2_O	n/a	20.5 μL
**Total**	n/a	50 μL
  c. Perform the PCR according to the manufacturer’s instructions. A representative example of the PCR condition is shown below.
    PCR cycling condition

**Table:** 

Steps	Temperature	Time	Cycles
Denaturation	98°C	10 s	20–35 cycles
Annealing	55°C	5 s	
Extension	72°C	5 s/kb	
Hold	4°C	Forever
    In the case of amplifying the ORF-containing DNA, a 20-cycles reaction is usually enough, as the 2^nd^ round PCR needs only a small amount of the DNA.

8. After the PCR, add 1 μL of DpnI to the PCR mixtures and incubate them at 37°C for 0.5–1.0 h to remove template pDNAs. In the case of a PCR to amplify 3′ UTR, this step can be ignored.
9. Mix a portion of the PCR mixtures (e.g., 2 μL) with loading dye and perform the electrophoresis using 1.2% agarose gels (100 V 25 min). Then, stain the gels with a gel-staining reagent (e.g., Midori Green Advance) and capture images of gels to confirm the size of the amplified DNAs.
10. Purify the amplified DNAs with a DNA purification kit according to the manufacturer’s instructions. For example, the Monarch PCR & DNA Cleanup Kit, but the DNA purification kit of your choice can also be used.
11. Quantify the concentration of the purified DNAs by absorbance using a spectrophotometer (a microvolume model such as NanoDrop 2000 is convenient).
12. 2^nd^ round PCR.
  a. To amplify IVT template DNAs containing T7 promoter, 5′ and 3′ UTRs, ORF, and poly(A) tail, prepare the PCR mixtures shown below. As in the case of the 1^st^ round PCR, you can also use another high-fidelity PCR enzyme of your choice.

**Table:** 1xMS2(U)site1

Reagent	Final concentration	Amount
PrimeSTAR Max Premix (2×)	1	25 μL
10 μM forward primer HNC-408	0.3 μM	1.5 μL
10 μM reverse primer HNC-396	0.3 μM	1.5 μL
3′ UTR PCR product	0.74 ng/μL (10 nM)	37 ng
The 1^st^ round ORF PCR product for 1xMS2(U)site1	0.02 ng/μL	1 ng
dH_2_O	n/a	Dilute to 50 μL
**Total**	n/a	50 μL

**Table:** 1xMS2(U)site2

Reagent	Final concentration	Amount
PrimeSTAR Max Premix (2×)	1×	25 μL
10 μM HNC-242	0.3 μM	1.5 μL
10 μM HNC-396	0.3 μM	1.5 μL
500 nM HNC-440	10 nM	1 μL
3′ UTR PCR product	0.74 ng/μL (10 nM)	37 ng
The 1^st^ round ORF PCR product for 1xMS2(U)site2 and 2xScMS2(C)-ORF	0.02 ng/μL	1 ng
dH_2_O	n/a	Dilute to 50 μL
**Total**	n/a	50 μL

**Table:** 2xScMS2(C)

Reagent	Final concentration	Amount
PrimeSTAR Max Premix (2×)	1×	25 μL
10 μM HNC-370	0.3 μM	1.5 μL
10 μM HNC-396	0.3 μM	1.5 μL
3′ UTR PCR product	0.74 ng/μL (10 nM)	37 ng
The 1^st^ round ORF PCR product for 1xMS2(U)site2- and 2xScMS2(C)-ORF	0.02 ng/μL	1 ng
dH_2_O	n/a	Dilute to 50 μL
**Total**	n/a	50 μL

**Table:** MS2CP-eDHFR, HaloTag-VPg(FCV), DD-CaVT, CaVT, or the control reporter

Reagent	Final concentration	Amount
PrimeSTAR Max Premix (2×)	1×	25 μL
10 μM HNC-242	0.3 μM	1.5 μL
10 μM HNC-396	0.3 μM	1.5 μL
500 nM KEC-62	10 nM	1 μL
3′ UTR PCR product	0.74 ng/μL (10 nM)	37 ng
The 1^st^ round ORF PCR product	0.02 ng/μL	1 ng
dH_2_O	n/a	Dilute to 50 μL
**Total**	n/a	50 μL
  b. Perform the PCR according to the manufacturer’s instructions. A representative example of the PCR condition is shown below.

**Table:** PCR cycling condition

Steps	Temperature	Time	Cycles
Denaturation	98°C	10 s	35 cycles
Annealing	55°C	5 s	
Extension	72°C	5 s/kb	
Hold	4 °C	Forever

13. Repeat steps 9–11 to confirm the size of the amplified DNAs, purify them, and measure their concentration. The concentration of purified IVT template DNAs should be higher than 90 ng/μL. If the concentration is too low, increase the PCR reaction volume or reduce the elution volume in the purification step.

**CRITICAL:** It is important to confirm the absence of extra bands by agarose gel electrophoresis. PCR by-products can be transcribed to unexpected RNAs. See troubleshooting 1 if extra bands are observed.

### Preparation of synthetic mRNAs by IVT

**Timing: 8–14 h**

This step describes the procedure to prepare synthetic mRNAs by in vitro transcription, followed by their dephosphorylation. The dephosphorylation step is necessary to reduce the immunogenicity of the mRNAs.

14. mRNA synthesis by IVT.
  a. Mix the components of the in vitro transcription reaction as shown below (10× T7 Reaction buffer, GTP, ATP, CTP, and T7 enzyme mix are components of MEGAscript T7 Transcription Kit). Note that the cap analogs of 1xMS2(U)site1 and site2 mRNAs are different from that of other mRNAs.

**Table:** 1xMS2(U)site1 and site2 mRNAs

Reagent	Final concentration	Amount
10× T7 Reaction buffer	1×	1 μL
G(5′)ppp(5′)A RNA Cap Structure Analog (100 mM)	6 mM	0.6 μL
GTP (75 mM)	1.5 mM	0.2 μL
ATP (75 mM)	7.5 mM	1 μL
CTP (75 mM)	7.5 mM	1 μL
N1-methyl-pseudoUTP (100 mM)	7.5 mM	0.75 μL
T7 enzyme mix	n/a	1 μL
Template DNA (1xMS2(U)site1 or site2)	40 ng/μL	400 ng
dH_2_O	n/a	Dilute to 10 μL
**Total**	n/a	10 μL

**Table:** Split CaVT, DD-CaVT, CaVT, the control reporter, and 2xScMS2(C) mRNAs

Reagent	Final concentration	Amount
10× T7 Reaction buffer	1×	1 μL
ARCA (100 mM)	6 mM	0.6 μL
GTP (75 mM)	1.5 mM	0.2 μL
ATP (75 mM)	7.5 mM	1 μL
CTP (75 mM)	7.5 mM	1 μL
N1-methyl-pseudoUTP (100 mM)	7.5 mM	0.75 μL
T7 enzyme mix	n/a	1 μL
Template DNA (Split CaVT, DD-CaVT, control reporter, or 2xScMS2(C))	40 ng/μL	400 ng
dH_2_O	n/a	Dilute to 10 μL
**Total**	n/a	10 μL
  b. Incubate the IVT reaction mixture at 37°C for 4–6 h.
    ***Note:*** We recommend using a constant-temperature incubator rather than a block heater for the IVT reaction. Incubation with a block heater for 4–6 h may cause water evaporation followed by condensation on the tube lid, which alters the concentration of the IVT reaction components.

15. Remove the template DNA by adding 1 μL of TURBO DNase (a component of MEGAscript T7 Transcription Kit) to each IVT reaction mixture and incubating at 37°C for 30 min.
16. Purify each mRNA using an RNA purification kit of your choice according to the manufacturer’s instructions (e.g., NEB Monarch RNA Cleanup Kit).
17. Dephosphorylate the mRNA using alkaline phosphatase (rApid alkaline phosphatase is given as an example) by mixing the components of the dephosphorylation reaction as shown below. Then, incubate the reaction mixture at 37°C for 30 min.

**Table:** 

Reagent	Final concentration	Amount
10× rApid alkaline phosphatase buffer	1×	4 μL
rApid alkaline phosphatase (1 U/μL)	25 mU/μL	1 μL
Purified mRNA	n/a	The whole eluted volume
dH_2_O	n/a	Dilute to 40 μL
**Total**	n/a	40 μL

18. Purify the mRNAs using an RNA purification kit according to the manufacturer’s instructions.
19. Measure the concentration of the purified mRNAs by absorbance spectroscopy.
20. Check the size and the quality of the purified mRNAs using Bioanalyzer and RNA 6000 pico kit according to the manufacturer’s instructions. Alternatively, other methods (e.g., Denaturing PAGE or Microchip Electrophoresis) could be used to analyze the sample purity and size.

**Figure 5:**
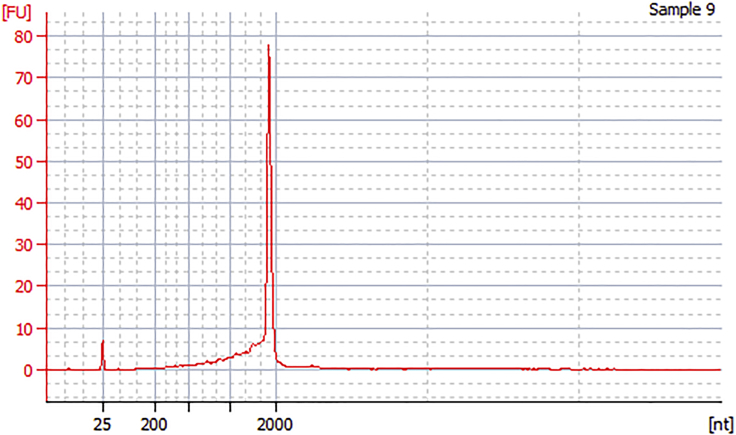
An example of an electropherogram analyzed by Bioanalyzer Most mRNAs should show a single peak, but 2xScMS2(C) mRNAs tend to show two peaks.

***Note:*** 2xScMS2(C) mRNA has a highly stable secondary structure, which is hard to denature, and can show two peaks. For the other mRNAs, only a single peak should be observed (Figure 5). See troubleshooting problem 2 if multiple peaks are observed.

### mRNA transfection, light irradiation, and expression analysis

**Timing: 3 days**

21. Seed the appropriate number of cells (e.g., 5 × 10^4^ HeLa cells in 500 μL/well of DMEM containing FBS and antibiotics) onto 24-well clear flat-bottom plates. Usually, 70%–90% confluent at transfection is suitable. To compare irradiated and non-irradiated conditions, at least two plates are needed. After seeding, incubate the cells at 37°C in a 5% CO_2_ incubator.

22. One day after the cell seeding, transfect the combinations of mRNAs shown in the table below. An example of a transfection procedure using Lipofectamine MessengerMAX is shown below.
  a. Prepare the diluted mRNA mixture in a sterile tube.

**Table:** Translational activation by split CaVT

Reagent	Amount
Opti-MEM	25 μL/well
1xMS2(U)site1 mRNA	320 ng/well
Control reporter mRNA	100 ng/well
MS2CP-eDHFR (N-terminal fragment of split CaVT) mRNA	20 ng/well
HaloTag-VPg(FCV) (C-terminal fragment of split CaVT) mRNA	60 ng/well

**Table:** Translational activation by DD-CaVT

Reagent	Amount
Opti-MEM	25 μL/well
1xMS2(U)site2 mRNA	360 ng/well
Control reporter mRNA	100 ng/well
DD-CaVT mRNA	40 ng/well

**Table:** Translational repression by DD-CaVT

Reagent	Amount
Opti-MEM	25 μL/well
2xScMS2(C) mRNA	80 ng/well
Control reporter mRNA	100 ng/well
DD-CaVT mRNA	40 ng/well
  b. Dilute the transfection reagent in a separate sterile tube.

**Table:** 

Reagent	Amount
Opti-MEM	25 μL/well
Lipofectamine MessengerMAX	1 μL/well
  c. Incubate for 10 min at room temperature.
    Prepare the transfection complex by mixing the diluted mRNA mixture and the diluted transfection reagent together and incubate for 5 min at room temperature.
  d. Add the transfection complex directly to the medium above the plated cells.
  e. Incubate the cells at 37°C in a 5% CO_2_ incubator for 3 h.
23. Prepare medium containing 250 nM photocaged TMP-HL (for split CaVT) or 10 μM photocaged TMP (for DD-CaVT). Medium containing TMP-HL or TMP without photocage can be used as a positive control. Avoid light irradiation to the photocaged ligands.
24. Three hours after the transfection, change the medium to the photocaged ligand-containing one. To avoid decaging of the ligands in the unirradiated control plate, shield the plate from light (e.g., by wrapping the plate with aluminum foil).
25. Place the cell culture plates directly onto an HP-30LM and irradiate with UV light (wavelength: 365 nm) from the bottom of the plates for 3–7 min. To avoid UV exposure to the experimenters, we recommend doing this procedure in a clean bench equipped with a UV shield. If a UV lamp other than HP-30LM is used for UV light irradiation, the irradiation time should be optimized depending on the light intensity. In the case of HP-30LM, the light intensity measured at the bottom of the plate by a photodiode power sensor was approximately 3.34 mW/cm^2^.
26. Incubate the cells at 37°C in a 5% CO_2_ incubator for 1 day.
27. Analyze the gene expression by a method suitable for the gene that is encoded by the transfected mRNA. An example of the procedure to analyze fluorescent protein expression using a flow cytometer is shown below.
  a. Detach the cells using 200 μL/well of 0.25% Trypsin/EDTA or other appropriate methods. Then, suspend the detached cells by adding 500 μL/well of the medium.
  b. Strain the cells using a cell strainer. Because the cells can aggregate over time, we recommend straining the cells immediately before measuring the fluorescence by flow cytometry.
  c. Measure the fluorescence by flow cytometry according to the manufacturer’s instruction.

***Note:*** Conditions to be tested are listed below.

**Table:** Translational activation by split CaVT

mRNA	Ligand	Light irradiation	Translation
▪ 1xMS2(U)site1 ▪ MS2CP-eDHFR ▪ HaloTag-VPg(FCV) ▪ Control reporter	Photocaged TMP-HL	+	Activated
▪ 1xMS2(U)site1 ▪ MS2CP-eDHFR ▪ HaloTag-VPg(FCV) ▪ Control reporter	Photocaged TMP-HL	-	Basal
▪ 1xMS2(U)site1 ▪ MS2CP-eDHFR ▪ HaloTag-VPg(FCV) ▪ Control reporter	TMP-HL	+ or -	Activated (positive control)
▪ 1xMS2(U)site1 ▪ MS2CP-eDHFR ▪ HaloTag-VPg(FCV) ▪ Control reporter	None	+ or -	Basal (negative control)
▪ 1xMS2(U)site1 ▪ MS2CP-eDHFR ▪ Control reporter	Photocaged TMP-HL or TMP-HL or None	+ or -	Basal (negative control, optional)
▪ 1xMS2(U)site1 ▪ HaloTag-VPg(FCV) ▪ Control reporter	Photocaged TMP-HL or TMP-HL or None	+ or -	Basal (negative control, optional)
▪ 1xMS2(U)site1 ▪ CaVT ▪ Control reporter	Photocaged TMP-HL or TMP-HL or None	+ or -	Activated (positive control, optional)

**Table:** Translational activation by DD-CaVT

mRNA	Ligand	Light irradiation	Translation
▪ 1xMS2(U)site2 ▪ DD-CaVT ▪ Control reporter	Photocaged TMP	+	Activated
▪ 1xMS2(U)site2 ▪ DD-CaVT ▪ Control reporter	Photocaged TMP	-	Basal
▪ 1xMS2(U)site2 ▪ DD-CaVT ▪ Control reporter	TMP	+ or -	Activated (positive control)
▪ 1xMS2(U)site2 ▪ DD-CaVT ▪ Control reporter	None	+ or -	Basal (negative control)
▪ 1xMS2(U)site2 ▪ Control reporter	Photocaged TMP or TMP or None	+ or -	Basal (negative control, optional)
▪ 1xMS2(U)site2 ▪ CaVT ▪ Control reporter	Photocaged TMP or TMP or None	+ or -	Activated (positive control, optional)

**Table:** Translational repression by DD-CaVT

mRNA	Ligand	Light irradiation	Translation
▪ 2xScMS2(C) ▪ DD-CaVT ▪ Control reporter	Photocaged TMP	+	Repressed
▪ 2xScMS2(C) ▪ DD-CaVT ▪ Control reporter	Photocaged TMP	-	Basal
▪ 2xScMS2(C) ▪ DD-CaVT ▪ Control reporter	TMP	+ or -	Repressed (positive control)
▪ 2xScMS2(C) ▪ DD-CaVT ▪ Control reporter	None	+ or -	Basal (negative control)
▪ 2xScMS2(C) ▪ Control reporter	Photocaged TMP or TMP or None	+ or -	Basal (negative control, optional)
▪ 2xScMS2(C) ▪ CaVT ▪ Control reporter	Photocaged TMP or TMP or None	+ or -	Repressed (positive control, optional)

***Optional:*** Analyze the data of the flow cytometry using appropriate software (*e.g.*, FlowJo).

## Expected outcomes

**Figure 6:**
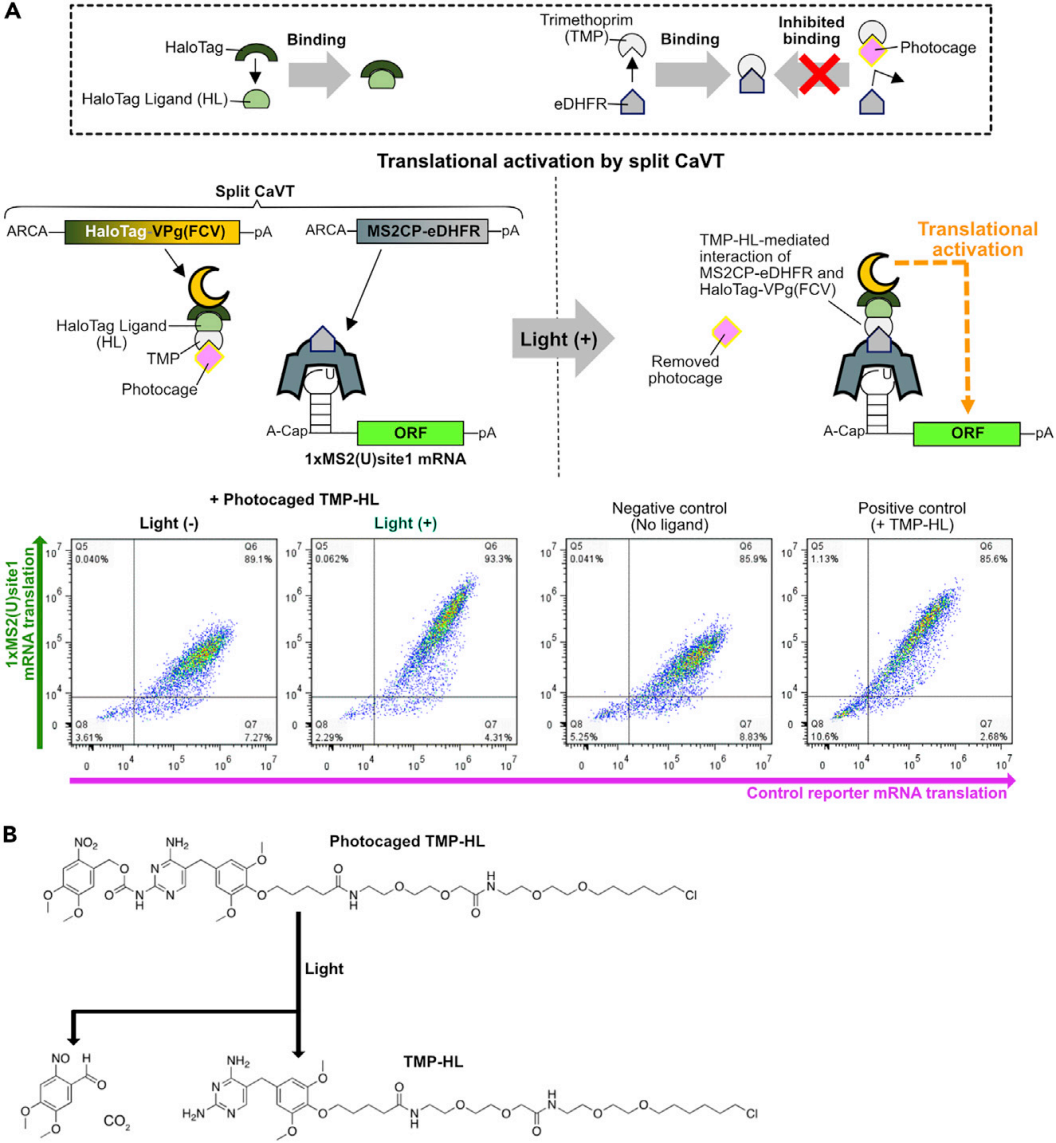
Light-induced translational activation by split CaVT (A) Schematic diagram and representative density plots. Split CaVT is composed of the N-terminal fragment (MS2 coat protein-*E. coli* dihydrofolate reductase, MS2CP-eDHFR) and the C-terminal fragment (HaloTag-VPg(FCV)). Light irradiation removes the photocage from the photocaged TMP-HL, which induces the formation of MS2CP-eDHFR-TMP-HL-HaloTag-VPg(FCV) complex. The complex binds 1xMS2(U)site1 mRNA and activates its translation. On the other hand, in the absence of light irradiation, the photocage prevents HaloTag-VPg(FCV) to interact with 1xMS2(U)site1 mRNA. To keep the basal translation level of 1xMS2(U)site1 mRNA low, it is capped with A-cap, a translationally inactive cap analog. (B) Photolysis of the photocaged TMP-HL.

**Figure 7:**
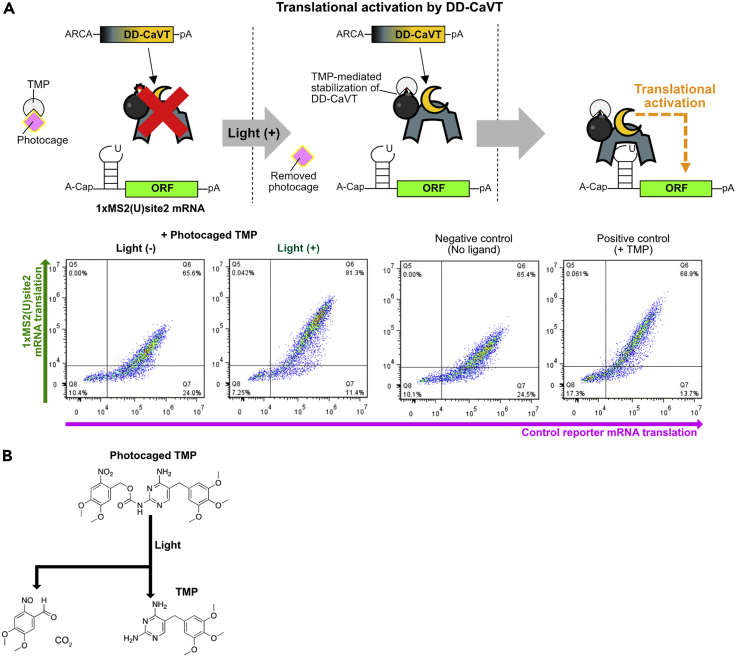
Light-induced translational activation by DD-CaVT (A) Schematic diagram and representative density plots. In the absence of light irradiation, DD-CaVT is rapidly degraded due to its destabilizing domain. Light irradiation removes the photocage from the photocaged TMP, which results in the stabilization of DD-CaVT by TMP. Then, the stabilized DD-CaVT translationally activates 1xMS2(U)site2 mRNA. Similar to 1xMS2(U)site1 mRNA in Figure 6, 1xMS2(U)site2 mRNA is capped with A-cap, a translationally inactive cap analog. (B) Photolysis of the photocaged TMP.

In the case of translational activation by split CaVT or DD-CaVT, cells treated with a photocaged ligand should show a light-dependent increase in the production of protein from 1xMS2(U)site1 or site2 mRNA (Nakanishi et al., 2021) (Figures 6 and 7).

**Figure 8:**
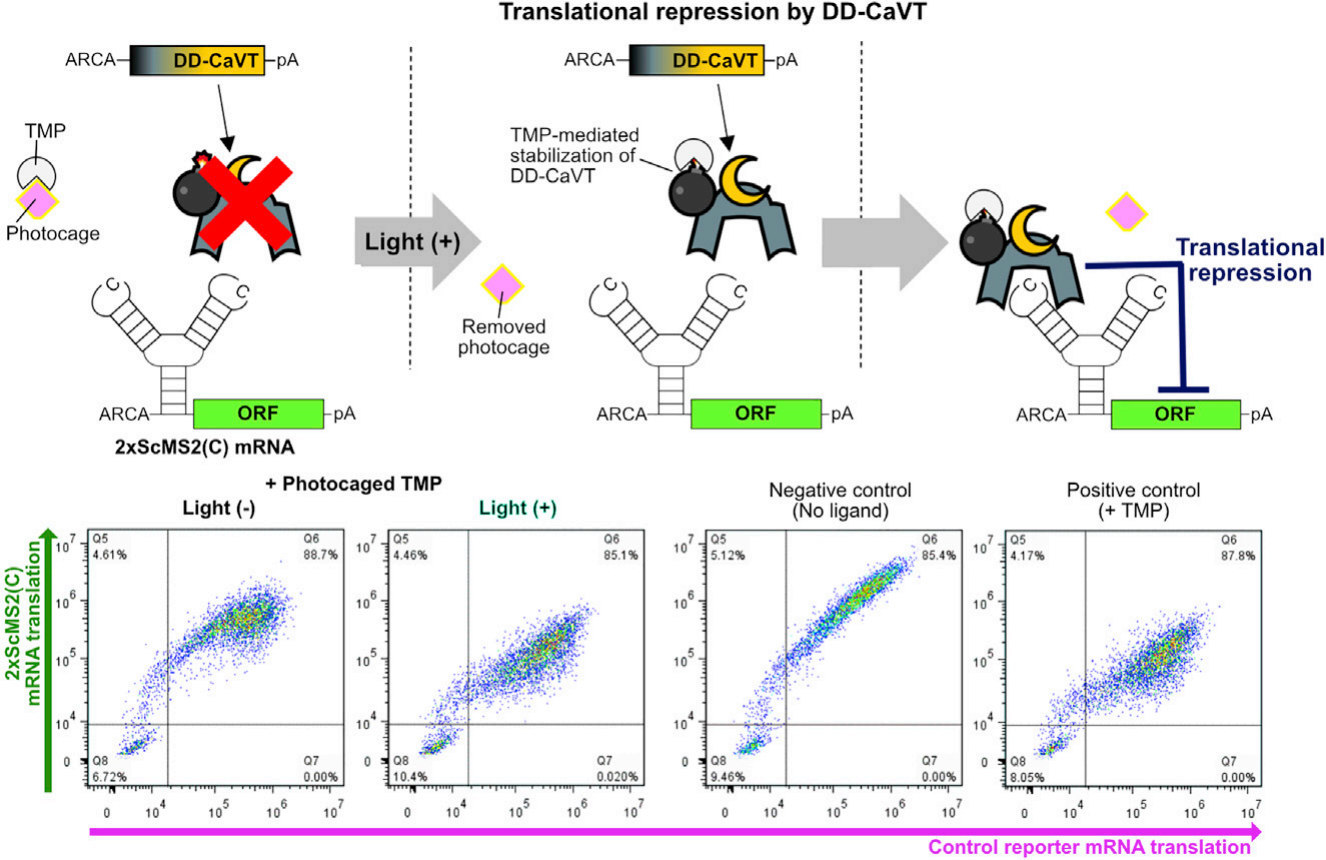
Schematic diagram and representative density plots of the light-induced translational repression by DD-CaVT Similar to the case of translational activation shown in Figure 7, the photolysis of the photocaged TMP stabilizes DD-CaVT. Then, the stabilized DD-CaVT binds 2xScMS2(C) mRNA. Different from the case of 1xMS2(U)site2 mRNA, the binding between 2xScMS2(C) mRNA and DD-CaVT is very strong, which results in translational repression rather than activation. To keep the basal translation level of 2xScMS2(C) mRNA high, it is capped with ARCA, a translationally active cap analog.

Conversely, in the case of translational repression by DD-CaVT, cells treated with a photocaged ligand should show a light-dependent decrease in the production of protein from 2xScMS2(C) mRNA (Figure 8).

## Limitations

Even in the translation-OFF state, there is usually leaky translation. Such leaky expression may affect cells even in the translation-OFF state should you want to regulate the mRNA encoding the protein with physiological activity. In addition, the expected fold-change by light irradiation is approximately three, which may be insufficient for some applications.

## Troubleshooting

### Problem 1

Extra bands are observed in the agarose gel electrophoresis of PCR products.

### Potential solution

Optimize the PCR conditions (e.g., annealing temperature, PCR enzyme, and ramp rate) or purify the main product using a DNA gel extraction and purification kit.

### Problem 2

Two or more peaks (in the case of 2xScMS2(C) mRNA, three or more peaks) are observed in the mRNA quality check by Bioanalyzer.

### Potential solution

Verify the absence of PCR by-products or residual pDNAs in the IVT template DNAs by running a larger amount of IVT template DNAs in the agarose gel electrophoresis. If PCR by-products are observed, optimize the PCR conditions, as described in troubleshooting problem 1. If residual pDNAs are observed, increase the reaction time or the enzyme concentration of the DpnI digestion. If only a single band is observed, check the RNA secondary structures by a secondary structure prediction tool, such as ParasoR (Kawaguchi and Kiryu, 2016) or MXfold2 (Sato et al., 2021). Stable stem-loop structures in mRNAs may cause multiple peaks even when the solution contains a single type of mRNA.

### Problem 3

Transfection efficiency is too low.

### Potential solution

Change the transfection condition. For example, extending the duration from transfection to medium change, using a transfection reagent other than Lipofectamine MessengerMAX (e.g., StemFect RNA Transfection Kit (ReproCELL) or TransIT-mRNA Transfection Reagent (Takara Bio)), or using an electroporator instead of a transfection reagent.

### Problem 4

The light-unirradiated group shows a similar translation level to the light-irradiated group and the positive control (a ligand without photocage-added) group. Only the negative control (no ligand addition) group shows a low (in the case of translation activation) or high (in the case of translational repression) level.

### Potential solution

The photocaged ligand may be uncaged due to light exposure during storage or there may be a failure in the caging reaction. Confirm the photocaged ligand by mass spectrometry. If the ligand is already uncaged, prepare a new lot of the photocaged ligand. To avoid the uncaging of the photocaged ligand, dispense and store it in a light-shielded condition.

### Problem 5

The light-irradiated group shows a similar translation level to the light-unirradiated group and the negative control (no ligand addition) group. Only the positive control (a ligand without photocage-added) group shows a high (in the case of translation activation) or low (in the case of translational repression) level.

### Potential solution

Increase the duration of the light irradiation.

### Problem 6

The positive control (a ligand without photocage-added) group shows a similar translation level to the negative control (no ligand addition) group.

### Potential solution

Co-transfect the target mRNA and the conventional (unsplit and no DD-fused) CaVT mRNA. If the conventional CaVT can translationally activate or repress the target mRNA translation, verify the quality and the preparation procedure of split CaVT or DD-CaVT. Even if the conventional CaVT cannot alter the target mRNA translation, verify the quality and the preparation procedure of the target mRNAs.

### Problem 7

The translation level can be regulated by light irradiation, but the absolute protein production is too low even in the translation-ON state.

### Potential solution

Except for the case of 1xMS2(U)site1 and site2 mRNAs, the absolute protein production may be improved using CleanCap AG reagent instead of ARCA. Note that CleanCap AG reagent needs the modified T7 promoter sequence (TAATACGACTCACTATAAGG) in IVT template DNAs instead of the usual T7 promoter sequence (TAATACGACTCACTATAGGG).

**Table:** 

Reagent	Final concentration	Amount
10× T7 Reaction buffer	1×	1 μL
CleanCap AG reagent (100 mM)	4.8 mM	0.48 μL
GTP (75 mM)	6 mM	0.8 μL
ATP (75 mM)	6 mM	0.8 μL
CTP (75 mM)	6 mM	0.8 μL
N1-methyl-pseudoUTP (100 mM)	6 mM	0.6 μL
T7 enzyme mix	n/a	1 μL
Template DNA	40 ng/μL	400 ng
dH_2_O	n/a	Up to 10 μL
**Total**	n/a	10 μL

Removal of double-stranded RNA by-products (Baiersdörfer et al., 2019) and optimization of the transfection conditions and codon usage may also improve absolute protein production. If you are using a target mRNA encoding a fluorescent protein, a brighter protein is also an option.

## Resource availability

### Lead contact

Further information and requests for resources and reagents should be directed to and will be fulfilled by the Lead and Technical Contacts, Hirohide Saito (hirohide.saito@cira.kyoto-u.ac.jp) and Hideyuki Nakanishi (nakanishi.hideyuki.3m@kyoto-u.jp).

### Materials availability

pDNAs necessary for split CaVT and DD-CaVT mRNA preparation can be obtained from Addgene. Other materials are commercially available.

## Data Availability

This study did not generate any datasets or codes.
